# Effect of insulin-like growth factor system on luteinising angiogenesis

**DOI:** 10.1530/RAF-22-0057

**Published:** 2023-04-21

**Authors:** Chinwe U Nwachukwu, Robert S Robinson, Kathryn J Woad

**Affiliations:** 1 School of Veterinary Medicine and Science, The University of Nottingham, Sutton Bonington campus, Loughborough, United Kingdom; 2 Department of Agricultural Science, School of Agriculture and Vocational Studies, Alvan Ikoku Federal College of Education, Owerri, Imo State, Nigeria

**Keywords:** insulin-like growth factors (IGFs), endothelial cells, IGF1 receptor inhibitor, angiogenesis, bovine

## Abstract

Preovulatory follicle growth and the luteal transition require intense angiogenesis. This enables progesterone production to increase sufficiently to support a pregnancy. Inadequate follicular or luteal vascularisation can lead to reduced ovarian function and thus compromise fertility. Insulin-like growth factor 1 (IGF1) and IGF2 regulate multiple ovarian processes and are key links between an animal’s reproductive and metabolic status. This study investigated the role that the IGF system plays in regulating luteinising follicular endothelial cell (EC) networks and progesterone production *in vitro*. Bovine luteinising follicular angiogenesis cultures were treated with (i) LR^3^-IGF1 (10 or 100 ng/mL) under basal and angiogenic-stimulated conditions or (ii) IGF1 receptor (IGF1R) inhibitor (picropodophyllin (PPP); 1 µM) in the presence or absence of LR^3^-IGF1, IGF2 or combined LR^3^-IGF1 + IGF2 (10 ng/mL). EC networks were quantified by von Willebrand factor immunohistochemistry. Progesterone production was analysed by ELISA, and cell proliferation was determined by MTT assay. LR^3^-IGF1 had limited effects on EC growth parameters, whilst PPP (*P* < 0.001) markedly reduced EC growth parameters (by 60–70%). Cell proliferation was slightly increased (by 3–5%) by LR^3^-IGF1 (*P* < 0.001). LR^3^-IGF1 had variable effects on progesterone production, whilst PPP reduced progesterone concentration (*P* < 0.001) with or without LR^3^-IGF1 or IGF2 alone or in combination. IGF1 was detected in cell-conditioned media and was increased by LH (50 ng/mL) (*P* < 0.001). In conclusion, exogenous IGF1 and IGF2 had minimal effects on luteinising follicular angiogenesis and progesterone production, but the inhibitory effect of the IGF1R inhibitor (PPP) suggests that IGF1R signalling is critical for the development of EC networks and progesterone production in luteinising follicular cells.

**Lay summary:**

The corpus luteum is a part of the ovary responsible for producing the critical pregnancy hormone, progesterone. To fulfil this function, the corpus luteum requires an extensive blood vessel network. Here, we investigated whether an important growth factor known to act on the ovary, insulin like growth factor (IGF) 1, critically regulates the formation of this blood vessel network and progesterone production. Cells from the corpus luteum were cultured with combinations of IGF1, a closely related hormone IGF2 and a chemical which stops both IGF1 and IGF2 from working. Afterwards, we measured the size and pattern of blood vessel networks, the production of progesterone and whether cells increased in number. We found adding IGF1 had limited effects, however stopping IGF1 from working had a very negative impact on both progesterone and on the formation of the blood vessel network. This suggests that cells from the corpus luteum were producing IGF1 and that IGF1/2 are critical for both blood vessel growth and hormone production.

## Introduction

The follicular–luteal transition is an extremely dynamic process involving numerous morphological and biochemical changes that occur after the luteinising hormone (LH) surge (Reynolds & Redmer 1999, Niswender *et al.* 2000). For these ovarian remodelling events to occur, the extensive growth of blood vessels and establishment of enhanced blood supply is crucial (Reynolds & Redmer 1999, Niswender *et al.* 2000). Furthermore, the rate of progesterone production is correlated to the ovarian blood flow and vascularisation (Fraser & Lunn 2001, Acosta *et al.* 2004, Schams & Berisha 2004). Hence, the inadequate function of the corpus luteum (CL) is considered a major cause of poor embryo development and early embryonic mortality in dairy cattle (Boland *et al.* 2001, Aco & Walsh 2011).

The best-characterised stimulators of angiogenesis are vascular endothelial growth factor A (VEGFA) and fibroblast growth factor 2 (FGF2) (Ferrara & Davis-Smyth 1997, Spicer & Aad 2007). Indeed, VEGFA and FGF2 are potent stimulators of angiogenesis in both follicular and luteal cells *in vitro* (Robinson *et al.* 2007, Woad *et al.* 2009, Laird *et al.* 2013). Moreover, local neutralization of FGF2 or VEGFA suppressed luteal growth and progesterone secretion during the early luteal phase in cattle (Yamashita *et al.* 2008). These studies demonstrate the critical importance of FGF2 and VEGFA for adequate luteal vascularisation and function. However, several other important growth factors have pro-angiogenic effects and include the insulin-like growth factor (IGF) system (Bach 2015, Van Beijnum *et al.* 2017, Dallinga *et al.* 2020). The IGF system plays an important regulatory role in the follicular–luteal transition and might be particularly relevant to the post-partum dairy cow, where circulatory IGF1 is nutritionally/metabolically regulated.

IGF1 and 2, like insulin, are pleiotropic hormones that regulate critical metabolic processes, cell proliferation, differentiation, migration, survival and apoptosis (LeRoith *et al.* 1995). Both IGF1 and IGF2 exert their actions through the IGF type 1 receptor (IGF1R), although IGF1 has a higher affinity for IGF1R than IGF2 (Federici *et al.* 1997, LeRoith 2003). IGF action is further regulated by six high-affinity IGF-binding proteins (IGFBP1–6) which modulate IGF bioavailability (LeRoith 2003, Clemmons 2007). IGF1 expression is controlled by various factors including nutrition and oestrogen (Wathes *et al.* 1995, Robinson *et al.* 2000), but its hepatic production is up-regulated by growth hormone (GH) (Friedrich *et al.* 2014) leading to elevated plasma IGF1 concentrations (Davis *et al.* 1992), Thus, plasma IGF1 concentrations can reflect the metabolic status of an animal. Indeed, heifers with negative energy balance (NEB) have lower plasma IGF1 concentrations which were associated with smaller CL and lower progesterone concentration than those with positive energy balance (Yung *et al.* 1996). In contrast, plasma IGF2 concentrations are much lower than IGF1 and are less GH-dependent in adult cattle (Lucy 2000).

IGF1 and IGF2 are also intra-ovarian growth factors that locally regulate follicular development (Webb & Campbell 2007). IGF1 was expressed by theca cells of bovine follicles throughout folliculogenesis, while in granulosa cells, *IGF1* mRNA expression increased after selection (Schams *et al.* 1999). *IGF2* mRNA was mainly localised to theca cells (Armstrong *et al.* 2000, Schams *et al.* 2002) with evidence that IGF2 is the major local intrafollicular IGF ligand regulating antral follicle growth in cattle. In the bovine CL, *IGF1* mRNA expression was greater in the mature CL compared with the developing CL (Woad *et al.* 2000). Using immunohistochemistry, IGF1 was localised mainly to large and small luteal cells with some staining evident in EC (Schams *et al.* 1999), while using *in situ* hybridisation, *IGF2* mRNA was expressed by steroidogenic luteal cells (Woad *et al.* 2000). IGF2 protein expression, however, was restricted to perivascular cells of large blood vessels and capillaries (Amselgruber *et al.* 1994).

*IGF1R* mRNA is present in theca and granulosa cells of dominant follicles (Schams *et al.* 1999). Acting through IGF1R, both IGF1 and IGF2 stimulates steroid production by follicular and luteal cells in many species (Spicer *et al.* 2000, Silva *et al.* 2009), in part, by up-regulating LH/CG receptor expression (Stewart *et al.* 1995, Campbell *et al.* 1998). IGF1 and IGF2 also promoted granulosa cell survival (Devoto *et al.* 1999, Sekar *et al.* 2000), proliferation and differentiation (Armstrong *et al.* 2000, Schams *et al.* 2002, Spicer & Aad 2007) in cows. In the bovine CL, *IGF1R* mRNA was abundantly expressed in the large and small luteal cells and in EC, albeit to a lesser extent (Woad *et al.* 2000). In luteal cells, IGF1 increased STAR protein expression and consequently had stimulatory effects on progesterone production (Devoto *et al.* 1999, Sekar *et al.* 2000).

In other tissues, intense IGF1R expression was detected in EC and pericytes of newly formed arteries and veins (Li *et al.* 2005), bovine aortic cells (Li *et al.* 2009) and human dermal microvascular endothelial cells (Chisalita & Arnqvist 2004). The role IGF1 plays in regulating angiogenesis is further demonstrated by IGF1 enhancing *de novo* vasculogenesis in human umbilical vein endothelial cells (HUVECs) (Friedrich *et al.* 2018), promoting proliferation and angiogenesis in rabbit retinal endothelial cells (Grant *et al.* 1993) and increasing the length and width of vessel-like structures in mouse brain endothelial cells (Lin *et al.* 2017). IGF1 can also indirectly promote ovarian angiogenesis, by increasing VEGFA expression in mouse granulosa cells (Martinez-Chequer *et al.* 2003) and bovine granulosa and luteal cells (Schams *et al.* 2001). Furthermore, inhibition of IGF1R signalling with picropodophyllin (PPP) reduced the formation of precursor EC in embryonic stem cells (Piecewicz *et al.* 2012), while IGF1R-targeted antibody completely blocked VEGFA-induced EC tube formation in HUVECs (Bid *et al.* 2012).

Collectively, this suggests that there is an active IGF system in the follicle and CL and that IGF1 and/or IGF2 have the potential to stimulate angiogenesis. However, there is very limited information on the role of IGF1 and/or IGF2 in controlling angiogenesis in the bovine ovary. This study thus hypothesised that IGF1 and/or IGF2, acting through the IGF1 receptor, will promote EC network formation and progesterone production in bovine luteinising follicular cells.

## Materials and methods

Bovine ovaries were collected from a local abattoir and transported back to the laboratory in phosphate-buffered saline (PBS) at room temperature. The collection and processing of the follicular cells were conducted as described by Laird *et al.* (2013). Briefly, large antral follicles (>10 mm, typically 8–10 per culture) with good vascularisation were dissected out and then hemisected. From these, granulosa cells were manually dispersed in a medium. The theca shells were enzymatically dispersed with 100 mg/mL collagenase type 1A, 50 mg/mL hyaluronidase and 0.2 units/mL DNase IV. The isolated granulosa and theca cells were washed three times in MCDB 131 media prior to the counting of viable cells and then plating. Granulosa cells (3 × 10^5^) plus theca cells (1 × 10^5^) to the final density of 4 × 10^5^ cells/well were plated onto fibronectin-coated coverslips in 12 well plates and allowed to luteinise. Cells were cultured in supplemented MDCB 131 media according to Laird *et al.* (2013) with the following important alterations: (i) LR^3^-IGF1 was omitted from the supplemented MDCB 131 media; (ii) insulin, transferrin and selenium (ITS) were added individually such that insulin was added at 100 ng/mL, rather than 10 µg/mL, thus minimising the potential stimulation of IGF1R by insulin; (iii) for experiments which studied the effects of LH on IGF1 production, LH was added as a treatment according to the experimental design. For all other experiments 5 ng/mL LH was added.

### The effect of IGF1 on angiogenesis and progesterone production in luteinising follicular cells

Bovine luteinising follicular cells (*n* = 4 cultures; duplicate wells per treatment) were cultured for 5 days and on day 1 were treated with either 0, 10 or 100 ng/mL LR^3^-IGF1 in the presence or absence of angiogenic stimulation (FGF2 + VEGFA; both 1 ng/mL). The doses of IGF1 were based on those used previously for ovarian cultures (Spicer & Aad 2007) and concentrations present in bovine circulation (Rhoads *et al.* 2008).

### Progesterone ELISA

Spent media were collected on days 3 and 5 and replaced with fresh supplemented MDCB 131 media. The collected media (from days 3 and 5) were stored at −20°C and subsequently analysed for progesterone concentration as an indicator of luteinisation over time. Progesterone analysis was performed in triplicate using a validated ELISA (Munro & Stabenfeldt 1984) following 100–200-fold dilution of samples in PBS. The samples from each culture were assayed in triplicate on a single plate and the mean intra-assay coefficient of variation (CV) was 14%.

### Immunocytochemistry

At the end of culture (day 5), the cells were fixed in ice-cold acetone:methanol (1:1) for von Willebrand factor (VWF) immunocytochemical analysis as previously described (Woad *et al.* 2012). Briefly, endogenous peroxidase activity was blocked with 3% hydrogen peroxide in methanol solution. After PBS washes, 20% normal goat serum (NGS) was added to reduce any non-specific binding. Next, the cells were incubated with polyclonal rabbit anti-human VWF (5 µg/mL; Dako: A0082) diluted in 2% NGS overnight at 4°C. On the next day, after PBS washes, the primary antibody was detected using the rabbit Elite ABC kit as per the manufacturer’s instructions and using 3,3'-diaminobenzidine (DAB) as the substrate (Vector Labs, Peterborough, UK).

### Image analysis

The EC networks were quantified by VWF immunocytochemistry and image analysis performed using Image Pro (Media Cybernetics) according toWoad *et al.* (2012). The four endpoints considered were (i) total area of EC networks; (ii) the number of EC networks present; (iii) the total number of branch points and (iv) the degree of branching, calculated as the number of branch points per EC network.

A sub-set of bovine luteinising follicular cells (*n* = 5 cultures) were cultured on fibronectin-coated 96 well plates. These cells were treated with control or 10 ng/mL LR^3^-IGF1, for 3 days in the absence of angiogenic stimulation; each treatment was performed in six replicates. On day 3, the cells were then prepared for the assessment of viable cell growth by utilising an MTT Cell Proliferation Assay (Berridge *et al.* 2005) as per manufacturer’s instructions (Cayman Chemical).

### The effect of LH on the production of IGF1 by luteinising follicular cells

Bovine luteinising follicular cells (*n* = 4 cultures, triplicate wells per treatment) were treated with different doses of LH (0, 5 or 50 ng/mL) (AFP11743B; biopotency 1.06 × oLH NIDDK-I-2; a gift from Dr A. F. Parlow, National Institute of Diabetes and Digestive and Kidney Diseases (NIDDK), Torrance, CA, USA).

### IGF1 ELISA

The spent media were collected on days 3 and 5 to determine IGF1 concentrations using a human IGF1 DuoSet ELISA, as per the manufacturer’s procedure (R&D Systems: DY291-05). Human and bovine IGF1 are identical in amino acid sequence. The samples from each culture were assayed on a single plate and mean intra-assay CV was 9.8%. The limit of detection was 50 pg/mL.

### Effect of IGF1R inhibition on angiogenesis and progesterone production by luteinising follicular cells

Bovine luteinising follicular cells (*n* = 4 cultures, duplicate wells per treatment) were treated from day 1 of culture with control, LR^3^-IGF1 (10 ng/mL), IGF2 (10 ng/mL), or combined LR^3^-IGF1+IGF2 in the presence or absence of a specific IGF1R inhibitor (1 µM; picropodophyllin (PPP); S7668; Selleckchem, Houston, TX, USA). For each culture (*n* = 4), each treatment was performed in duplicate. PPP is widely recognised as a specific and selective inhibitor of IGF1R tyrosine kinase signalling with no known activity at the insulin receptor, FGFR, PDGFR or EGFR (Girnita *et al.* 2004). There are some reports that PPP potentially has some IGF1R-independent actions by targeting p53 (Liao *et al.* 2021). The dose of PPP was based on its IC50, the doses previously used in similar culture systems and minimising any off-target effects (Larsson & Axelson 2007, Bieghs *et al.* 2014).

PPP was initially dissolved in DMSO, with the final DMSO concentration in each treatment being 0.1%. Thus, the appropriate control wells were treated with 0.1% DMSO. The cells were treated throughout culture with the experiment completed on day 5. Spent media were collected on days 3 and 5 of culture and analysed for progesterone production. On day 5 of culture, the cells were fixed in ice-cold acetone:methanol, and the EC network formation was quantified by VWF immunohistochemistry and image analysis.

### Statistical analysis

Data were checked for normality using residual plots and for homogeneity of variance with Levene’s test; where appropriate, data were log-transformed. For all experiments, *P* < 0.05 was declared as significant and all experimental data values are presented as mean ± s.e.m. from replicated cultures.

For the EC network parameters (i.e. total EC network area, degree of branching number of branch points) and cell proliferation, the data were converted to % of the mean for the control wells for that particular culture. The data were then analysed using a randomised-block ANOVA using Genstat 20th Edition (Hemel Hempstead, United Kingdom). For each endpoint, the data were blocked by culture, with the particular well nested within. The exact factors depended on the factorial design of each study. Namely, (i) for the effect of IGF1 on EC network formation, a two-way ANOVA was performed with IGF1 and presence of angiogenic stimulation as the factors and (ii) for the effect of IGF1R inhibition on EC network formation, a three-way ANOVA was performed with IGF1, IGF2 and PPP as the factors. The main effects and their interaction were included in each statistical model.

For the progesterone production analysis, data were log-transformed where appropriate. The day of culture was initially kept in the factorial design. However, as predicted, there was a large effect of culture day and thus the data from days 3 and 5 were analysed independently. Thus (i) for the effect of IGF1 on progesterone concentrations, a two-way ANOVA was performed with IGF1 and presence of angiogenic stimulation as the factors; and (ii) for the effect of IGF1R inhibition on progesterone, a three-way ANOVA was performed with IGF1, IGF2 and PPP as the factors.

Initially, when determining the effect of LH on IGF1 concentrations, the day of culture was incorporated into a two-way factorial design. However, there was a day effect and thus for each day independently a one-way randomised-block ANOVA was used with the factor being LH treatment. Student–Newman–Keuls multiple comparison test determined which doses of LH were different.

## Results

### The effect of IGF1 on angiogenesis and progesterone production in luteinising follicular cells

**Figure 1:**
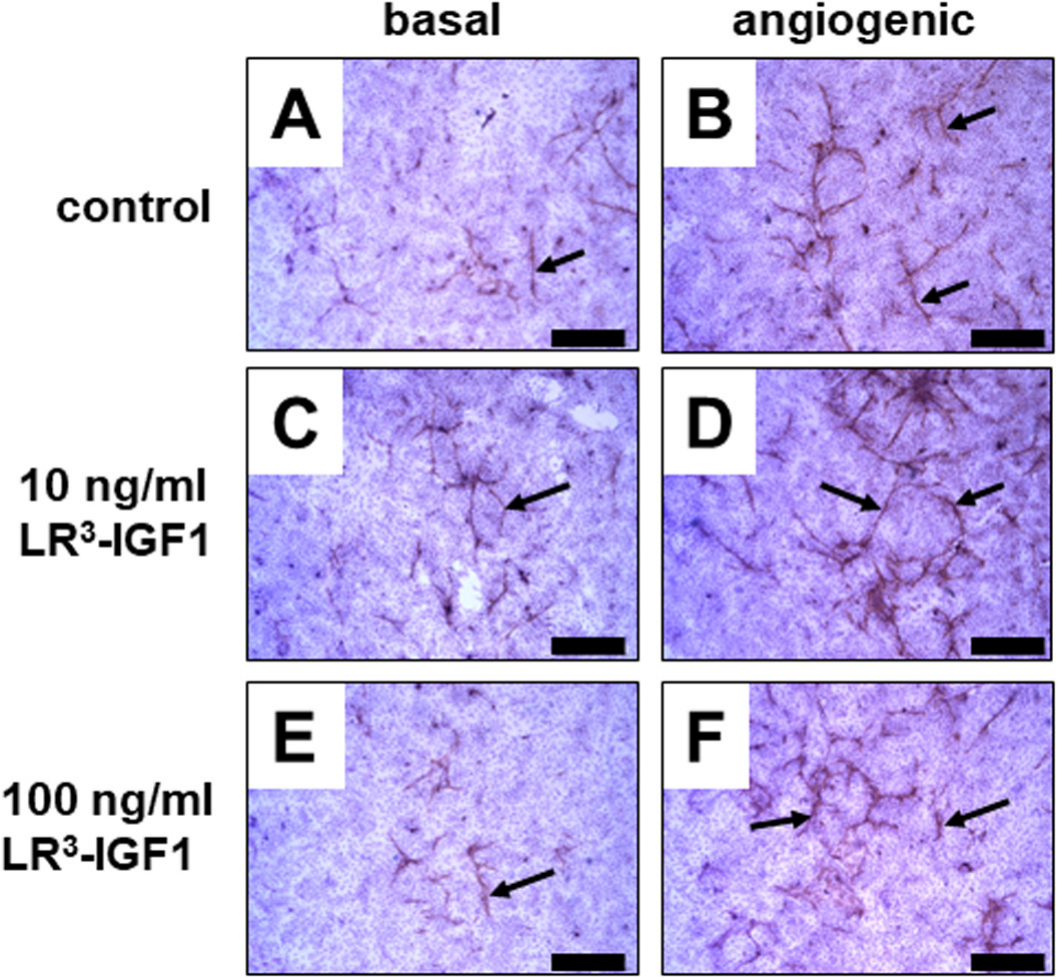
The effect of LR^3^-IGF1 on endothelial cell (EC) network formation in the absence (A, C, E; basal) and presence (B, D, F; angiogenic) of FGF2 + VEGFA-stimulation in bovine luteinising follicular cells. Cells were treated with either 0 (A, B), 10 (C, D) or 100 ng/mL LR^3^-IGF1 (E, F). The EC networks were identified with von Willebrand factor immunocytochemistry as indicated by the positive brown staining (arrows). Scale bars represent 500 µm.

**Figure 2:**
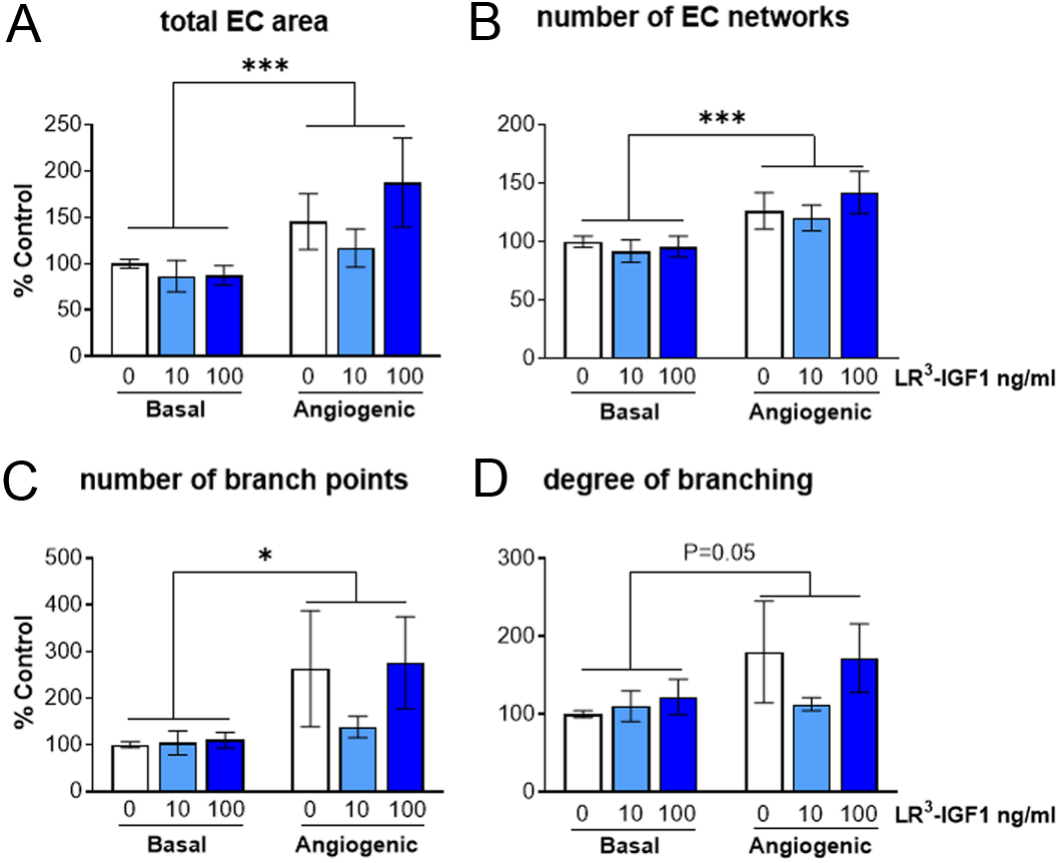
Effect of LR^3^-IGF1 on endothelial cell (EC) network formation in the absence (basal) and presence (angiogenic) of FGF2 + VEGFA in bovine luteinising follicular cells. LR^3^-IGF1 was added at 0, 10 or 100 ng/mL in both the absence and presence of FGF2 + VEGFA (both 1 ng/mL). The effects of treatment are shown on (A) total area of EC networks, (B) number of EC networks, (C) number of branch points and (D) degree of branching. LR^3^-IGF1 treatment had no effect (*P* > 0.05) on total area and number of EC networks, number of branch points and degree of branching. However, the addition of FGF2 + VEGFA increased (***P < 0.001) the area and number of EC networks by 30% and the number of branch points (*P < 0.05). The data are mean ± s.e.m.; *n* = 4 cultures.

In the control wells, endothelial cells (EC) were closely associated with each other and were largely organised into structures with a network-like appearance. These EC networks (a group of closely apposed ECs) were often elongated and tubule-like in appearance, with some complex multiple branching points extending from the central point of the EC network. The vast majority of the ECs formed into a network-like pattern, but some ECs were observed in small islands with limited branching extending from the central body of cells. Other cell types (presumably steroidogenic cells, pericytes and smooth muscle cells) were more numerous and present alongside the ECs (Fig. 1A, B and C). The growth and development of the EC networks appeared to be greater under angiogenic-stimulated conditions (FGF2 + VEGFA; Fig. 1B, D and E). Indeed, angiogenic stimulation with FGF2 + VEGFA treatment increased the total area of EC networks (by 50% of basal; *P* < 0.001), number of EC networks (by 30% of basal; *P* < 0.001) and number of branch points (by 200% of basal; *P* < 0.05) (Fig. 2A, B and C). However, the degree of branching (*P* = 0.05) only tended to be increased by angiogenic stimulation (Fig. 2D and F).

The organised, intricate EC networks were also present following LR^3^-IGF1 treatment in the presence and absence of angiogenic stimulation (Fig. 1C, D, E and F). There was a similar pattern of EC networks, in terms of number, shape and size, across all LR^3^-IGF1 treatments. Quantification demonstrated that LR^3^-IGF1 treatment at either dose had no effect on the total area of EC networks (*P* > 0.05), the number of EC networks (*P* > 0.05), the number of branch points (*P* > 0.05) or the degree of branching (*P* > 0.05; Fig. 2A, B, C and D). In addition, this effect was the same under basal and angiogenic stimulation and there was no LR^3^-IGF1 by FGF2+VEGFA treatment interaction (*P* > 0.05).

**Figure 3:**
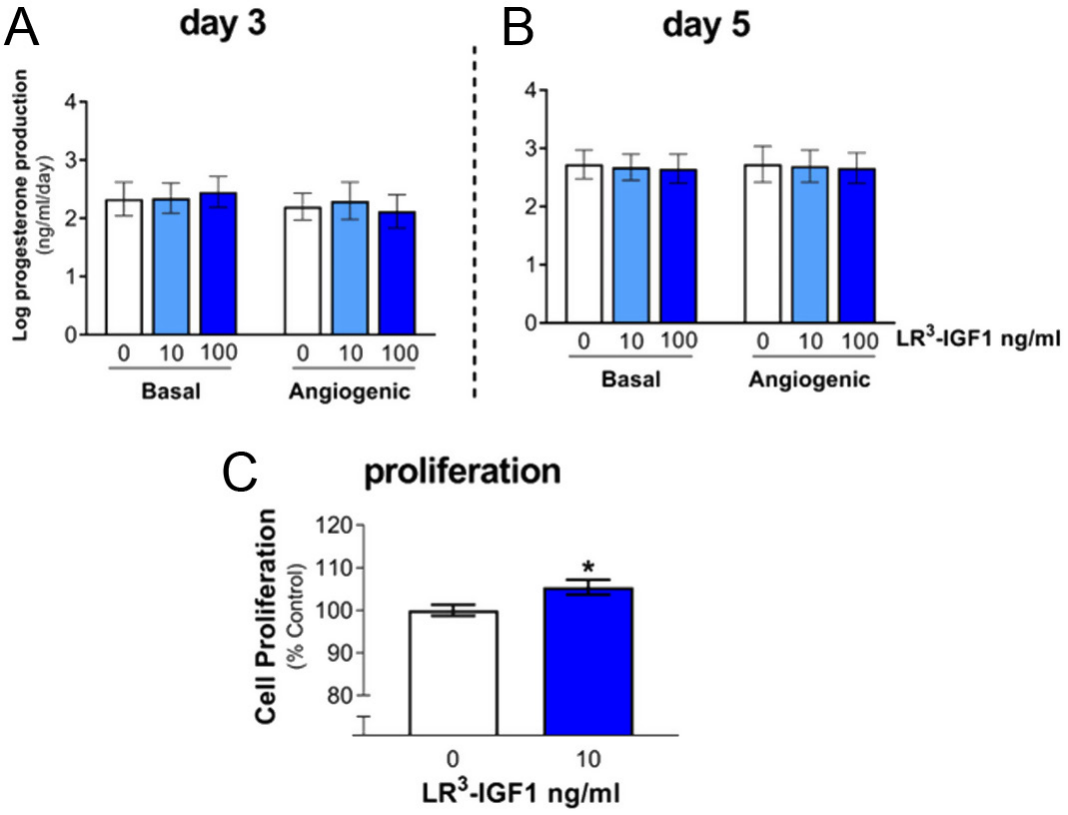
Effect of LR^3^-IGF1 on progesterone production by bovine luteinising follicular cells on (A) day 3 and (B) day 5 of culture in the absence (basal) and presence (angiogenic) of FGF2 + VEGFA (both 1 ng/mL). Progesterone production (ng/mL/day) increased between days 3 and 5 (*P* < 0.001), whilst LR^3^-IGF1 treatment (*P* > 0.05) had no effect on progesterone production. Angiogenic stimulation tended (*P* = 0.05) to decrease progesterone concentration on day 3 but not on day 5. The data are mean ± s.e.m.; *n* = 4 cultures. (C) shows that LR^3^-IGF1 increased the proliferation of bovine luteinising follicular cells after 72 h in culture (* vs control; *P* < 0.05). The data (mean ± s.e.m.; *n* = 5 culture) are expressed as a percentage of the control cells.

Progesterone production increased over time in culture, with progesterone concentrations in the spent media being 2.5-fold greater on day 5 than day 3 (*P* < 0.001; Fig. 3). Angiogenic stimulation (FGF2 + VEGFA) tended to slightly decrease progesterone concentration (*P* = 0.05) on day 3 but had no effect on day 5 (*P* > 0.05). Progesterone production was unaffected by LR^3^-IGF1 treatment on day 3 of culture (*P* > 0.05; Fig. 3A) and day 5 of culture (*P* > 0.05; Fig. 3B). Additionally, there was no LR^3^-IGF1 by FGF2 + VEGFA treatment interaction (*P* > 0.05). Cell proliferation was slightly increased by LR^3^-IGF1 treatment (*P* < 0.05; Fig. 3C).

### The effect of LH on the production of IGF1 by luteinising follicular cells

**Figure 4:**
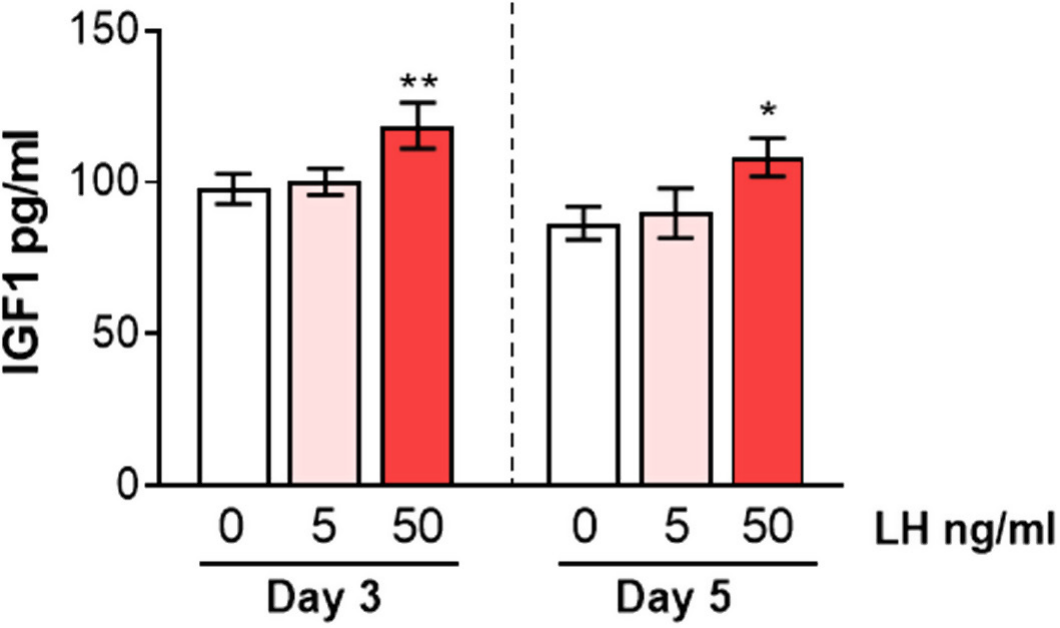
The endogenous IGF1 production by bovine luteinising follicular cells and the effect of LH treatment. LH treatment (50 ng/mL) increased IGF1 concentration in conditioned media on both day 3 and day 5 of culture. The data are mean ± s.e.m.; *n* = 4 cultures; *P < 0.05 and **P < 0.01 vs respective control.

Next, the level of endogenous production of IGF1 was determined, to investigate whether this could explain the lack of IGF1 treatment effect; for this study, no exogenous IGF1 or LR^3^-IGF1 was added. IGF1 was detected in the spent media of luteinising follicular cells at 80–120 pg/mL (Fig. 4) with IGF1 concentrations decreasing with time in culture (*P* < 0.01). On each day of culture, LH at 50 ng/mL, but not at 5 ng/mL, increased IGF1 concentrations compared with the control. This was by 21% on day 3 (*P* < 0.01) and by 24% on day 5 (*P* < 0.05; Fig. 4).

### Effect of IGF1R inhibition on angiogenesis and progesterone production by luteinising follicular cells

This experiment investigated whether IGF1R signalling was involved in the formation of EC networks and progesterone production in bovine luteinising follicular cells. In addition, since IGF2 also acts via IGF1R, it was important to determine what effects IGF2 exerted. Thus, the effects of IGF1R inhibition in the presence and absence of exogenously added LR^3^-IGF1, IGF2 and combined LR^3^-IGF1 plus IGF2 treatments were investigated.

**Figure 5:**
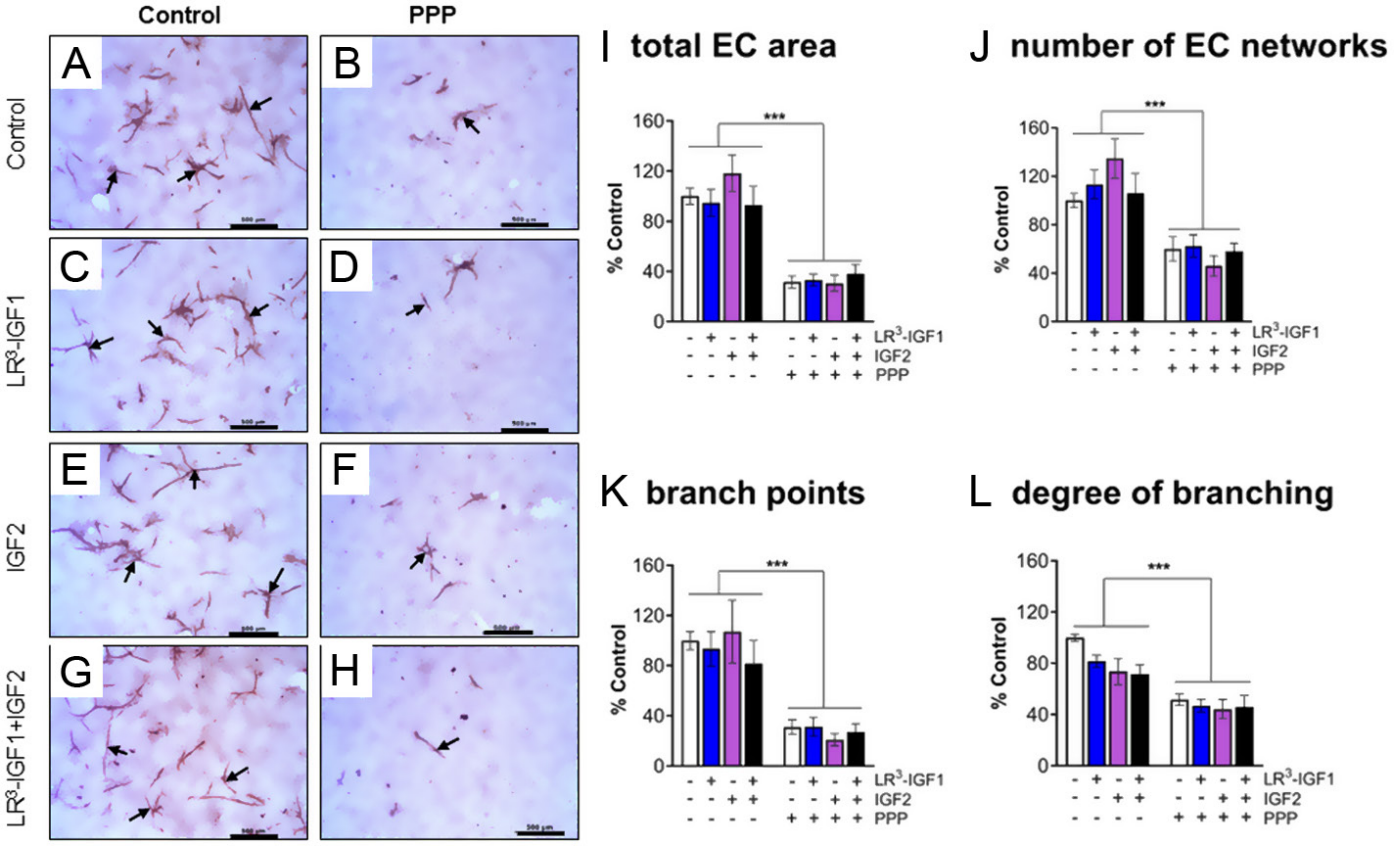
Effect of inhibition of IGF1R signalling on endothelial cell (EC) network formation in bovine luteinising follicular cells under basal conditions. Representative images show EC network formation in the absence (A, C, E, G) and presence (B, D, F, H) of the IGF1R inhibitor, picropodophyllin (1 µM PPP). Cells were treated with either 0 (A, B), 10 ng/mL LR^3^-IGF1 (C, D), 10 ng/mL IGF2 (E, F) or 10 ng/mL LR^3^-IGF1+IGF2 (G, H). The EC networks were immunostained with von Willebrand factor as indicated by the positive (brown) staining (arrows) of the EC networks. The scale bars represent 500 µm. The quantification of the treatment effects on (I) total area of EC networks, (J) number of EC networks, (K) number of branch points and (L) degree of branching is shown. PPP treatment reduced all EC network endpoints by 60–70%. Treatment with LR^3^-IGF1 or IGF2 had no effect on the majority of EC network parameters, except for the degree of branching which was overall slightly reduced by IGF2 (*P* < 0.05). The data values are mean ± s.e.m. from four cultures. ***P < 0.001 vs no PPP treatment.

By the end of the culture, the cells were confluent across the whole of each coverslip; however, the density of cells appeared to be slightly reduced in the IGF1R inhibitor-treated wells. Highly organised, intricate EC networks were formed in control wells in the absence of IGF1R inhibitor (PPP; Fig. 5). However, it was clearly evident that in the PPP-treated wells, there were fewer EC networks and those that were present were smaller and had reduced branching. Indeed, treatment with PPP had marked negative effects on all EC network endpoints measured. Namely, PPP treatment (*P* < 0.001) reduced the total area (*P* < 0.001; Fig. 5I), number of EC networks (*P* < 0.001; Fig. 5J), number of branch points (*P* < 0.001; Fig. 5K) and degree of branching (*P* < 0.001; Fig. 5L) by 60–70% vs untreated wells. Visibly, the apparent inhibitory effect of PPP on the size and appearance of the EC networks was similar in those cells treated with IGF1, IGF2 or control (Fig. 5). Quantification confirmed that this reduction was observed in both the presence and absence of exogenous LR^3^-IGF1, IGF2 and combined LR^3^-IGF1 + IGF2 treatments (Fig. 5). There was no effect of LR^3^-IGF1 or IGF2 treatment on most EC network endpoints that were measured; the number (*P* > 0.05), total area (*P* > 0.05) and number of branch points (*P* > 0.05). However, IGF2 slightly reduced the degree of branching in both the absence and presence of PPP (*P* < 0.05; Fig. 5L). There were no LR^3^-IGF1 by IGF2 interactions (*P* > 0.05; Fig. 5).

**Figure 6:**
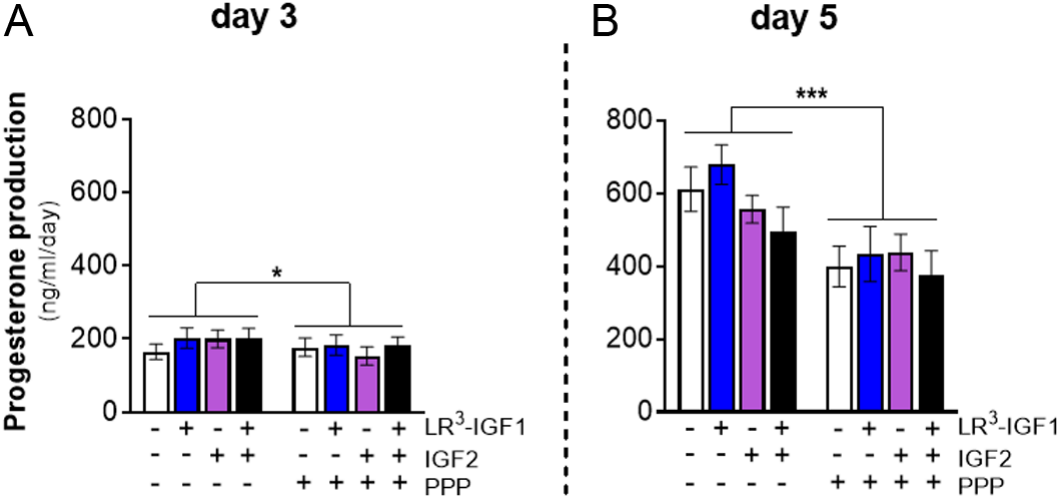
Effect of inhibiting IGF1R signalling on progesterone production in control and IGF-treated bovine luteinising follicular cells on (A) day 3 and (B) day 5 of culture. Progesterone production was affected by the day of culture (*P* < 0.001) with nearly 3-fold higher production on day 5. PPP decreased progesterone production on days 3 (*P < 0.05) and 5 (***P < 0.001). On day 3 of culture, LR3-IGF1, overall, increased progesterone production (*P* < 0.05) but had no effect on day 3. In contrast, IGF2 decreased progesterone production (*P* < 0.05) on day 5 of culture. The data values are mean ± s.e.m. (*n* = 4 cultures).

Next, the effect of PPP on progesterone production was determined. As expected, progesterone concentrations were 2.7-fold greater on day 5 than on day 3 of culture (*P* < 0.001, Fig. 6). For subsequent analysis of any treatment effects, progesterone production was analysed on each culture day separately. On day 3 of culture, overall, LR^3^-IGF1 treatment (*P* < 0.05) slightly increased progesterone production by 11% (Fig. 6A), while IGF2 had no effect on progesterone production (*P* > 0.05; Fig. 6A). On day 5 of culture, LR^3^-IGF1 had no effect, but IGF2 (*P* < 0.05) overall decreased progesterone production by 12% (Fig. 6B). Overall, PPP slightly decreased progesterone production on days 3 (by 10%; *P* < 0.05) and 5 of culture (by 30%; *P* < 0.001; Fig. 6) irrespective of IGF treatment. There were no LR^3^-IGF1 or IGF2 by PPP treatment interactions on day 3 (*P* > 0.05; Fig. 6A). However, on day 5 of culture, there was an LR^3^-IGF1 by IGF2 interaction (*P* < 0.05) indicating that progesterone production was reduced when LR^3^-IGF1 and IGF2 were combined (*P* < 0.05; Fig. 6B).

## Discussion

This study tested the hypothesis that IGF1 and/or IGF2 acting through IGF1R promotes EC network formation and progesterone production in bovine luteinising follicular cells. This culture system was selected since it mimics the follicular–luteal transition and enables EC networks to develop. This is the first time that the involvement of IGF1R signalling in the stimulation of angiogenesis and progesterone production in luteinising follicular cells has been investigated. Contrary to expectations, LR^3^-IGF1 or IGF2 treatment failed to increase the extent of EC network formation or progesterone production. However, when IGF1R activation was inhibited, EC network formation and progesterone production were markedly reduced. This indicates that IGF1R signalling has a profound influence on EC and luteal cell function. It also suggests that the lack of LR^3^-IGF1 and IGF2 effect was due to the endogenous stimulation of IGF1R by local IGFs and the activation of IGF1R-induced angiogenesis and steroidogenesis.

In two different experiments, LR^3^-IGF1 treatment had no effects on the formation of EC networks which was contrary to the original hypothesis. This also disagrees with previous studies in other tissues where IGF1 dose-dependently stimulated the migration of bovine carotid artery ECs (Nakao-Hayashi *et al.* 1992, Piecewicz *et al.* 2012) and EC tube formation in HUVECs (Bid *et al.* 2012). In the present study, two doses of LR^3^-IGF1 treatment were included (10 and 100 ng/mL) and these were based on plasma IGF1 concentrations in cows during periods of NEB and positive energy balance (Kobayashi *et al.* 1999, Fenwick *et al.* 2008). Additionally, IGF1 and IGF2 at 100 ng/mL increased the proliferation of bovine granulosa cells (from large follicles) by 1.6- to 1.8-fold (Nakao-Hayashi *et al.* 1992, Spicer & Aad 2007). Thus, it is unlikely that the lack of an IGF effect was due to doses being inappropriate. Additionally, in the present study, LR^3^-IGF1 (at 10 ng/mL) stimulated follicular cell proliferation as determined by MTT assay demonstrating that this dose was efficacious. Furthermore, the dose of LR^3^-IGF1 used was determined to be 100–1000-fold higher than the mean concentration of IGF1 (0.1 ng/mL) detected in the spent media.

There is much evidence suggesting that the IGFs act in synergy with VEGFA and FGF2 (Sun *et al.* 2009). Thus, the action of IGF1 on luteinising follicular cells under basal or angiogenic-stimulated conditions was tested. While angiogenic stimulation (FGF2 plus VEGFA) increased the number of EC networks formed and the total EC network area by 30%, there was no interaction with IGF1. Under both basal and angiogenic-stimulated conditions, IGF1 had no effect, which indicated that there was no synergistic or additive effect between the IGF1 and the pro-angiogenic growth factors.

One explanation for the lack of IGF1 and IGF2 effects on EC network formation is that there was high endogenous production of IGF1 and/or IGF2 in the follicular culture system. Hence, the concentration of IGF1 was measured in the spent media and whether this was affected by LH. Indeed, luteinising follicular cells endogenously produce IGF1 in concentrations of 100 pg/mL. This concentration is considerably below the estimated EC50 for IGF1 (0.4–50 ng/mL) in various cell types (Duleba *et al.* 1997, Punglia *et al.* 1997, Gustafsson *et al.* 1999). Moreover, IGF1 spent media concentrations were 100-fold lower than those used as treatments and significantly below the levels found in follicular fluid (100-200 ng/mL) and plasma (10-100 ng/mL) (Ortega *et al.* 2008, Rodríguez *et al.* 2017). Collectively, this strongly indicates that any endogenous production of IGF1 would not have been great enough to override the exogenous LR^3^-IGF1 treatment. Interestingly, LH, at similar levels to that observed during the LH surge, increased IGF1 production in luteinising follicular cells. This could be a mechanism by which gonadotropins and growth factors synergise to stimulate EC development during the follicular–luteal transition. We were unable to determine the presence of IGF2 in the spent media as there was no bovine-specific IGF2 ELISA available; thus, it is feasible that endogenous IGF2 production by follicular cells could exert these effects. In support of this possibility, IGF2 mRNA and protein expression has been localised to the bovine CL (Amselgruber *et al.* 1994, Woad *et al.* 2000). An alternative explanation is that insulin, acting through IGF1R is masking the action of the IGF ligands. While feasible, the concentration of insulin was reduced 100-fold from 10 µg/mL present in ITS supplement to 100 ng/mL to minimise any insulin-induced activation of IGF1R signalling (Li *et al.* 2005). However, further studies are required to eliminate this as a possibility.

Based on the lack of IGF1 effects, it was important to investigate the role that IGF1R signalling plays in regulating endothelial and luteal cell function. The approach taken was to target IGF1R with a specific tyrosine kinase inhibitor, PPP, which affected EC network formation in other systems (Larsson & Axelson 2007, Bieghs *et al.* 2014). In this present study, PPP treatment markedly reduced (60–70%) EC network formation compared to controls. Specifically, PPP reduced total area and number of EC networks, plus the number of branch points and degree of branching. These inhibitory effects were similar in the absence or presence of exogenous LR^3^-IGF1, IGF2 or combined LR^3^-IGF1 and IGF2 treatment. This clearly indicated that IGF1R signalling has a profound effect on angiogenesis in bovine luteinising follicular cells. This is in agreement with previous findings where IGF1R-targeted antibodies reduced EC tube formation in HUVECs (Bid *et al.* 2012) and IGF1R antagonist attenuated ischaemia-induced retinal neovascularisation by inhibiting VEGF signalling (Smith *et al.* 1999). There is strong evidence indicating that IGF1R is expressed in multiple cell types in the pre-ovulatory follicle and CL including endothelial cells (Woad *et al.* 2000, Schams *et al.* 2002). Thus, while IGF1R could directly influence endothelial cell function, it is most likely to stimulate angiogenesis by promoting VEGFA and/or FGF2 action.

### Progesterone production: effect of IGF1 and IGF2

Progesterone production increased over time in culture, which indicated that the follicular cells were undergoing luteinisation and/or proliferating. However, the effect of IGF1 treatment on progesterone production was varied, with one experiment showing a small increase while the other revealed no effect. Similarly, a limited effect of IGF1 on progesterone production has been reported in human granulosa cells that were undergoing luteinisation (Foster *et al.* 1995). However, in most other reports, IGF1 (and IGF2) stimulated progesterone production in follicular and luteal cells across many animal species (Scaramuzzi *et al.* 1999, Spicer & Aad 2007, Silva *et al.* 2009). The reason for this discrepancy is unknown, but it is feasible that in the present study, the steroidogenic cells are undergoing luteinisation and steroidogenesis at near their maximal capacity such that there is limited potential for this to be increased. Indeed, Laird *et al.* (2013) showed that in the same culture system, LH increased progesterone production but only by 15%. Thus, it is feasible that as there are a number of stimulants present that induce follicular luteinisation (e.g. fibronectin coating, presence of LH and other exogenous growth factors), the effects of such treatments are masked by the already high level of progesterone synthesis.

Progesterone production, though, was markedly decreased by IGF1R inhibition with PPP and the degree of this suppression increased with time in culture. Specifically, by day 5 of culture, progesterone concentrations were 60% lower in cells treated with PPP. Thus, IGF1R signalling plays an important role in stimulating the production of progesterone. This could be further explored by elucidating the interplay of IGFs with the insulin receptor, which is also known to promote steroidogenesis in follicles (Spicer & Echternkamp 1995).

The IGF system is an important link between the metabolic status of an animal and its reproductive function (Lucy 2000). For example, inadequate nutrition and metabolic imbalances down-regulate reproductive function in ruminants. Indeed, alterations in circulatory metabolic hormones (e.g. GH, insulin and IGF1) are associated with reduced fertility (Wathes *et al.* 2003). NEB results in reduced CL weight and decreased progesterone concentration in the blood and/or milk of lactating cows (Butler 2000, Leroy *et al.* 2008). Additionally, NEB during the peripartum period affects serum IGF1 concentrations (Diskin *et al.* 2003) and may affect follicular development, the follicle–luteal transition and subsequent luteal function (Spicer *et al.* 2000). Indeed, reduced serum IGF1 concentrations were associated with poor luteal growth and progesterone production in cows (Burns *et al.* 1997). Therefore, diminished IGF signalling could limit luteal angiogenesis during the follicle–luteal transition, with a critical impact on luteal development and function.

## Conclusion

In conclusion, exogenously added IGF1 and IGF2 had minimal effects on luteinising follicular angiogenesis and progesterone production, but the inhibitory effect of the IGF1R inhibitor (PPP) suggests that IGF1R signalling was critical for the development of EC networks and progesterone production in luteinising follicular cells. Thus, this study highlights the importance of IGF signalling in regulating luteal angiogenesis and function in the cow.

## Declaration of interest

The authors declare that there is no conflict of interest that could be perceived as prejudicing the impartiality of the research reported.

## Funding

This work was supported by Tetfund Nigeria.

## Author contribution statement

RSR & KJW conceived the study, CUN performed experiments, RSR analysed data, CUN, RSR & KJW wrote the paper.
